# GSTT1, GSTP1, and GSTM1 genetic variants are associated with survival in previously untreated metastatic breast cancer

**DOI:** 10.18632/oncotarget.22450

**Published:** 2017-11-14

**Authors:** Jian Zhang, Ying Wu, Xichun Hu, Biyun Wang, Leiping Wang, Sheng Zhang, Jun Cao, Zhonghua Wang

**Affiliations:** ^1^ Department of Medical Oncology, Fudan University Shanghai Cancer Center, Department of Oncology, Shanghai Medical College, Fudan University, Shanghai 200032, China

**Keywords:** GSTT1, GSTP1, GSTM1, polymorphism, metastatic breast cancer

## Abstract

**Purpose:**

The polymorphisms in genes including GSTM1, GSTP1 and GSTT1 have been found to predict development and therapeutic efficacy in various malignancies. Breast cancer is one of most common cancers among women. In this study, we evaluated the prognostic value of three functional polymorphisms of GSTs in patients with previously untreated metastatic breast cancer (MBC).

**Patients and Methods:**

The genotype of GSTT1, GSTP1, and GSTM1 in 170 patients with previously untreated MBC from one single center were assessed via PCR-based RFLP methods. The prognostic of polymorphisms on overall survival (OS) was examined using the Kaplan-Meier estimates and Cox proportional hazard ratio (HR) regression analyses.

**Results:**

The null genotypes of GSTT1 and GSTM1 were significantly correlated to poor OS compared with the present genotypes, respectively. After adjusting for clinic-pathologic factors, GSTT1 and GSTM1 genetic variants were still significantly associated with OS (HR, 1.92; 95% CI, 1.26-2.91 and HR, 1.53; 95% CI, 1.05-2.23). GSTT1 and GSTM1 were independent survival predictors and GSTP1 was not associated with overall survival of previous untreated MBC.

**Conclusion:**

This exploratory analysis suggests that in addition to clinic-pathologic factors, the genetic variants in GSTT1 and GSTM1 might be predictive of survival outcome in patients with previously untreated MBC.

## INTRODUCTION

Breast cancer is a heterogeneous disease and the most common cancer among women with a drastically increasing rate in China [1]. Approximately 6% of women were initially diagnosed with metastatic breast cancer (MBC) and about 20% of patients would develop to MBC at an early stage [2]. Despite of significant improvements in the treatment of MBC during the last decade, it still remains an incurable disease with a median overall survival of 18-30 months [3]. Current therapy decision of MBC relies on clinical features, histological factors and well-defined biomarkers [4]. Effective chemotherapy drugs used in the treatment of various malignant tumors always result in drug resistance and toxicity. It was reported that many genetic polymorphisms were involved in metabolism enzyme function, drug resistance, toxicity and efficiency of chemotherapy [5–11]. Hunting for genetic markers to improve clinical outcome of MBC patients becomes a big challenge.

Glutathione S-transferase (GST) enzymes play an indispensable role in detoxifying chemotherapy drugs. They detoxify products of oxidation or alkylating drugs by directly combining to reactive compounds or drugs [12, 13]. GSTT1(glutathione S-transferase theta), located on chromosome 22, plays a role in human carcinogenesis. GSTP1(glutathione S-transferase pi), located on chromosome 11, prevents cells from carcinogen and cytotoxin. And GSTM1(glutathione S-transferase mu), located on chromosome 1, is involved in detoxification and drugs action [14, 15]. Studies have shown that GSTs gene polymorphisms might aid to identify high-risk individuals of chronic diseases such as type 2 diabetes mellitus (T2DM), hypertension and so on [16–18]. In addition, GSTs genetic variants have been reported to be involved in fluorouracil and platinum-based chemotherapy of various metastatic or advanced cancers, such as acute myeloid leukemia, gastrointestinal tumor, non-small cell lung cancer and prostate cancer [19–21].

For potential prognostic value, genetic polymorphism is also important for patients with MBC. Moreover, genetic polymorphism can be easily detected and applied to clinical application. Because genetic polymorphism is found to be strongly associated with chemotherapy efficacy and prognosis of breast cancer, it can be used to establish a refined model to predict prognosis of this disease [22]. Therefore, we performed a study in patients with previously untreated MBC to assess the impact of GSTM1 null/present, GSTT1 null/present, and GSTP1 rs1695 polymorphisms on the survival. The present study demonstrates that these genetic polymorphisms in MBC cancer patients have the potential to be developed as novel biomarkers for diagnosis and prognosis of MBC patients.

## RESULTS

### Patient characteristics and clinical outcomes

The distribution of demographic, treatment characteristics and clinical features of patients are presented in Table 1. By the time of the final analysis (December 2012), the median follow-up time of the patients was 35.8 months. One hundred and twenty-eight patients (75.3%) died and the median survival time was 21.6 months [95% confidence interval (CI), 18.6-24.6 months]. The 1-, 3-, and 5-year OS rates were 82. 4%, 65.9%, and 16.9%, respectively.

**Table 1:** Patient clinical and treatment characteristics

Characteristics	N (%)
Age at MBC diagnosis (years)	
Median	50. 0
Range	25. 0-74. 0
≥ 60	28 (16. 5)
40-59	113 (66. 5)
< 40	29 (17. 1)
Menstruation status	
Post-menopausal	71 (41. 8)
Pre-menopausal	99 (58. 2)
Molecular subtype	
Luminal A	36 (21. 2)
Luminal B (HER-2 negative)	12 (7. 1)
HER-2 positive	18 (10. 6)
Triple-negative	98 (57. 6)
Unknown	6 (3. 5)
Adjuvant therapy	
No	18 (10. 6)
Only CT (± RT)	119 (70. 0)
CT + HT (± RT)	33 (19. 4)
Relapse-free interval	
Median (months)	15. 2
≤ 2 years	119 (70. 0)
> 2 years	51 (30. 0)
No. of metastatic sites	
1	69 (40. 6)
2	45 (26. 5)
≥3	56 (32. 9)
Metastatic site^*^	
Liver	50 (29. 4)
Lung	75 (44. 1)
Brain	6 (3. 5)
Lymph node	108 (63. 5)
Bone	45 (26. 5)
Chest wall	32 (18. 8)
Others	38 (22. 4)
Type of metastatic site	
Non-visceral	51 (30. 0)
Visceral	119 (70. 0)

Abbreviation: MBC, metastatic breast cancer; CT, chemotherapy; RT, radiotherapy; HT, hormone therapy; No., number.

^*^ Number determined at MBC diagnosis includes metastasis to more than one site

### Comparison of survival according to baseline characteristics of patients

To test whether various clinical characteristics contribute to overall survival (OS), patients were grouped according to age, menstruation status, molecular subtype, previous adjuvant treatment, RFI, number of metastatic sites, and type of metastatic site. According to univariate analysis, age, RFI, number of metastatic sites, and type of metastatic site significantly influenced patient prognosis (Table 2).

**Table 2:** Univariate and multivariate Cox-regression analyses for OS

Genotype	Univariate analysis		Multivariate analysis	
	HR (95% CI)	*P*	HR (95% CI)	*P*
Age at MBC diagnosis				
≥ 60	1. 00			
40-59	1. 90 (1. 12-3. 20)	0. 017		
< 40	2. 34 (1. 25-4. 37)	0. 008		
Menstruation status				
Post-menopausal	1. 00			
Pre-menopausal	1. 28 (0. 90-1. 81)	0. 176		
Molecular subtype				
Luminal A	1. 00			
Luminal B (HER-2 negative)	0. 92 (0. 45-1. 88)	0. 817		
HER-2 positive	1. 30 (0. 82-2. 05)	0. 271		
Triple-negative	1. 28 (0. 68-2. 40)	0. 446		
Adjuvant therapy				
No	1. 00			
Only CT (± RT)	0. 70 (0. 40-1. 21)	0. 201		
CT + HT (± RT)	0. 72 (0. 38-1. 36)	0. 310		
Relapse-free interval				
≤ 2 years	1. 00		1. 00	
> 2 years	0. 58 (0. 39-0. 86)	0. 007	0. 56 (0. 38-0. 84)	0. 005
No. of metastatic sites				
1	1. 00			
2	1. 01 (0. 65-1. 56)	0. 983		
≥3	1. 63 (1. 09-2. 46)	0. 019		
Type of metastatic site				
Non-visceral	1. 00		1. 00	
Visceral	1. 80 (1. 20-2. 72)	0. 005	1. 68 (1. 11-2. 55)	0. 015
GSTT1 deletion	1. 84 (1. 22-2. 76)	0. 003	1. 92 (1. 26-2. 91)	0. 002
GSTM1 deletion	1. 43 (1. 00-2. 04)	0. 046	1. 53 (1. 05-2. 23)	0. 028

Abbreviation: OS, overall survival; MBC, metastatic breast cancer; CT, chemotherapy; RT, radiotherapy; HT, hormone therapy; No., number.

### Effects of SNPs on OS

**Figure 1:**
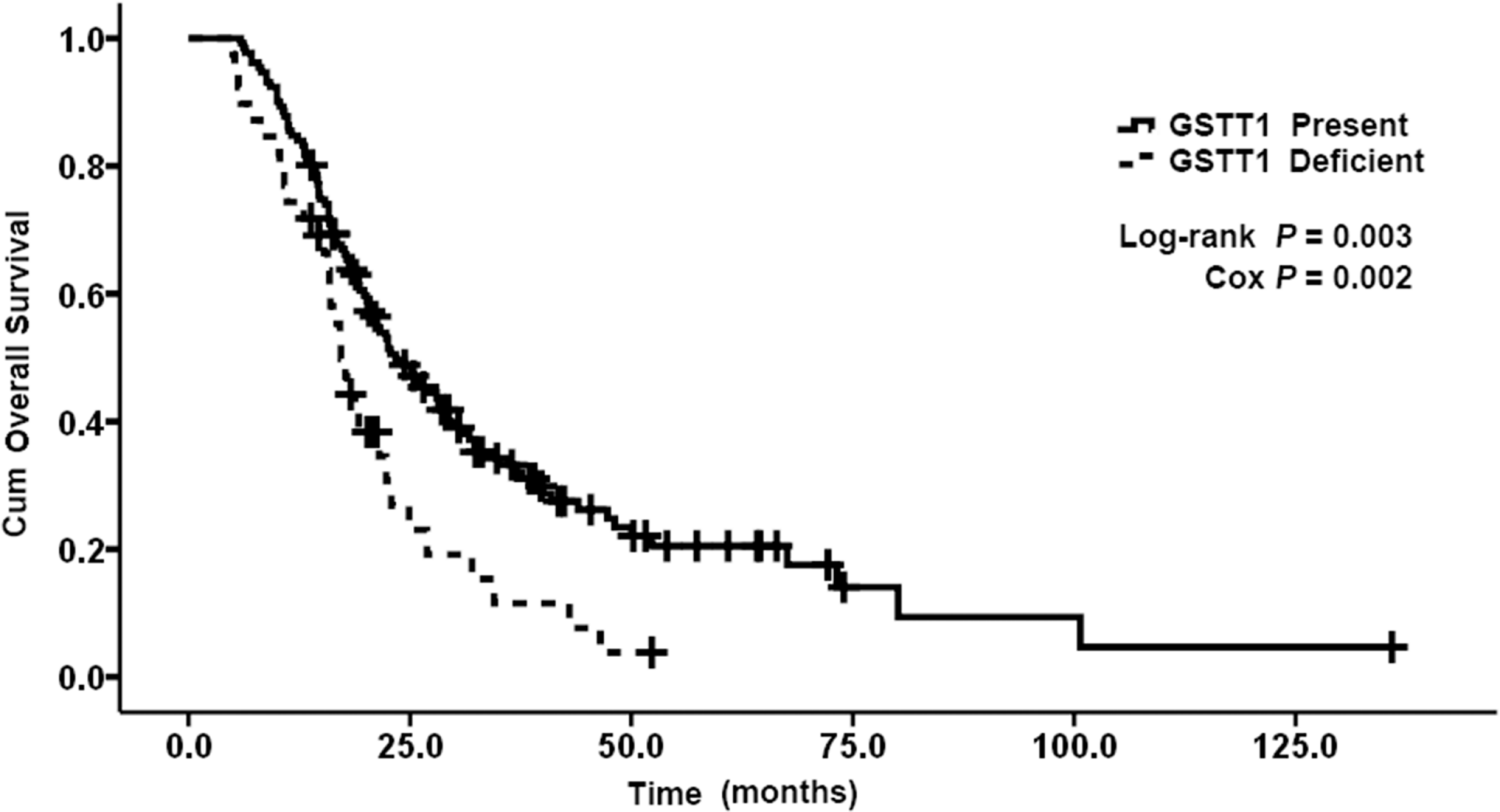
Kaplan–Meier curve demonstrating the overall survival (OS) of genotypes of GSTT1 The median OS was 23. 4 months (95% CI: 18. 7-28. 1) in present genotypes of GSTT1 and 17. 2 months (95% CI: 14. 9-19. 5) in null genotypes of GSTT1; p=0. 003.

**Figure 2:**
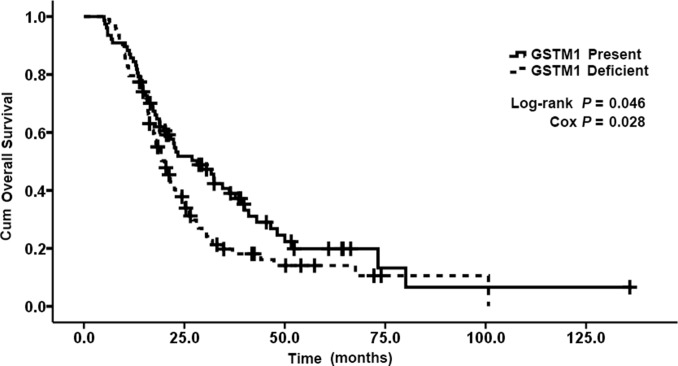
Kaplan–Meier curve demonstrating the overall survival (OS) of genotypes of GSTM1 The median OS was 28. 2 months (95% CI: 18. 8-37. 6) in present genotypes of GSTM1 and 20. 1 months (95% CI: 17. 0-23. 2) in null genotypes of GSTM1; p=0. 046.

The allelic frequencies for multiple genes’ variants are summarized in Table 3. All observed genotype frequencies in patients were verified to be consistent with the HWE. Interestingly, the GSTT1, GSTM1 polymorphisms were significantly associated with patient survival. As shown in Table 3, patients with the present genotypes of GSTT1 and GSTM1 had 6.2 and 8.1 months longer survival (median OS, 23.4 and 28.2 months; 95% CI, 18.7-28.1 and 18.8-37.6 months, respectively) than those with the null genotypes (median OS, 17.2 and 20.1 months; 95% CI, 14.9-19.5 and 17.0-23.2 months, respectively; P = 0.003 and 0.046 for log-rank test; Figures 1 and 2). But GSTP1 rs1695 was not found associated with overall survival of previously untreated MBC in our study.

**Table 3:** Associations between genotypes and OS

Genotype	No.	OS			
		Median (mo)	*P*	HR	95%CI
GSTT1					
Present	131	23. 4		1. 00	
Null	39	17. 2	0. 003	1. 84	1. 22-2. 76
GSTM1					
Present	77	28. 2		1. 00	
Null	93	20. 1	0. 046	1. 43	1. 00-2. 04
GSTP1 (rs1695)					
AA	116	20. 4		1. 00	
AG	50	22. 9	0. 768	1. 06	0. 72-1. 57
GG	4	50. 0	0. 292	0. 47	0. 12-1. 91
AG+GG	54	24. 3	0. 992	1. 00	0. 68-1. 46

Abbreviation: No., number; mo, month; OS, overall survival; HR, hazard ratio; CI, confidence interval; NC, not calculated.

### Multivariate analysis

In the multivariate Cox proportional hazards model, after adjustment for age, menstruation status, molecular subtype, previous adjuvant treatment, or number of metastatic sites, the prognostic significance of GSTT1, GSTM1 polymorphisms, RFI and type of metastatic site still existed. The hazard ratios (HRs) of patients with GSTT1 null genotype, GSTM1 null genotype, RFI > 2 years or visceral metastasis on OS were 1.92 (95% CI, 1.26-2.91), 1.53 (95% CI, 1.05-2.23), 0.56 (95% CI, 0.38-0.84) and 1.68 (95% CI, 1.11-2.55), respectively (Table 2).

## DISCUSSION

As an incurable disease, MBC need systemic treatments which include chemotherapy, endocrine therapy, molecular therapy and immunotherapy. Some clinical characteristics are fundamental for therapy decision, such as lymph node metastasis, hormone receptors status, human epidermal growth factor receptor 2 (HER2) expression and types of metastatic site [23]. Molecular targeting therapies and immunotherapy have shown important and potential status in recent years for their remarkable effect and lower toxicity compared with the traditional chemotherapies. The discovery and use of agents targeted to ER, PR and HER2 have provided clinician with effective therapies. However, drug-resistance remains a crucial obstacle to tackle [24]. What is more, the potential of biomarker-based treatments improving target therapies, emphasized the requirement to find molecular markers involved in pathogenesis of breast cancer, which are the prognostic factors of therapeutic response and survival [25].

A number of prognostic factors have been shown to significantly predict the survival of patients with metastatic disease. These mainly include adjuvant chemotherapy, RFI, dominant metastatic site, menopausal status, receptor status, and multiple organ involvement [26]. In terms of the metastatic site, visceral like liver diffusion was reported to be a predictor of undesirable survival while non-visceral metastatic including only metastatic in bony skeleton or a single bone lesion can be considered as an indolent disease [27, 28]. Genetic polymorphisms involving in drug metabolism, DNA repair and apoptosis could alter the efficacy of chemotherapeutic regimens, and hence have effects on cancer progression.

In the present study, we examined the association of GSTs genetic polymorphisms and patient survival in a cohort of 170 patients with previously untreated MBC. We found that the null genotypes of GSTT1 and GSTM1 significantly contributed to poorer OS compared with the present genotypes, respectively. After adjusting for clinic-pathologic factors, the two genetic variants were still significantly associated with OS, showing that these polymorphisms were independent survival predictors. Additionally, RFI and type of metastatic site were also independently associated with OS of MBC patients in the cohort.

The glutathione-S-transferases (GSTs) make up a family of multifunctional enzymes with detoxification ability on electrophilic compounds [29]. In some previous clinical studies, the higher levels of GST enzymes in tumors are considered to reduce responses to chemotherapy and associated with a poorer survival in patients with carcinomas of the breast [30], stomach [31], esophagus [32], ovary [33, 34] and head and neck [35]. It has been identified that independent gene deletion are unable to express an active protein at both GSTM1 and GSTT1 [36, 37]. One might expect that a null genotype of enzyme would increase response to chemotherapy and improve clinical outcome [13]. Recently. Jian et al. [38] examined GST genotypes in 244 advanced non-small cell lung carcinoma patients, they found the null GSTM1 and the GG genotype of GSTP1 IIe105Val were correlated improved overall survival. Tatjana I. Djukic et al. [39] found patients with increased level of GSTT1 enzymes has the shorter mean life expectancy compared to null GSTT1.

Our results are in accordance with previous reports that null genotypes of GSTT1 and GSTM1 could be a poorer prognosis and showed a sluggish response to chemotherapy in various types of cancers [40–44]. To our knowledge, only one study has investigated the association between polymorphisms in the GST genes and OS in previously untreated patients with MBC [45]. Similar to our result, no associations were found between GSTP1 rs1695 and progression free survival [45]. It should be pointed out that, the results from early breast cancer (EBC) have been mixed. Sweeney et al. [46] and Ambrose et al. [47] found that the early stage patients with the low-activity GSTP1 Val/Val genotype and both null genotypes of GSTM1 and GSTT1 have better OS after chemotherapy. Whereas no effectiveness was reported for GSTP1 [48], GSTM1 [49], and GSTT1 [49] in other studies.

Thus, this is the first study to find the null genotypes of GSTT1 and GSTM1 significantly contribute to poorer OS compared with the present genotypes in MBC, which is quite different from the results in EBC setting [46, 47], but in accordance with the findings in other metastatic cancer types [40–44]. This may be partly because reduced GST activity leads to increased glutathione levels and elevated glutathione reduced the capability of DNA to bind to cytotoxic drugs such like platinum compounds [42, 50, 51], DNA-reactive metabolites of anthracyclines, and various alkylating agents. These most commonly used chemotherapeutic agents in the treatment of breast carcinoma, are substrates for GST-mediated glutathione conjugation [52, 53], more than that, the glutathione can protect DNA from damage and adduct formation by coupling [54]. That is why these enzymes are susceptible to chemotherapy. The function of GSTs extends beyond detoxification and chemosensitivity, as they have been found to play a critical role in kinase signaling [55, 56]. The function of cell signaling controlled will provide novel therapeutic targets of new drugs. It will provide the possibility to develop antagonists or agonists aiming at signaling pathway and exert positive biological effect. Additional, acquiring of GSTs genotyping from blood samples leads to personalized modality and better effectiveness. Therefore, the variants of GSTT1 and GSTM1 could be a novel and helpful predictive factor to identify specific MBC patients who may benefit from signaling pathway. As our study is the inclusion of untreated MBC, the identified patients may acquire higher response and lower toxicity as they accept targeting therapies earlier, at the same time, spare those patients unlikely to benefit from needless therapies and toxicity.

Several limitations of this study should be noted. The sample size of our study is relative moderate. And further research is necessary to choose patients according to genetic characteristics and find the optimized targeted treatment or tailored chemotherapy for patients with null genotypes of GSTT1 and GSTM1. Results of the presented study should be validated in prospective studies. And, due to possible ethnic differences, our results should be further verified in different ethnic populations to acquire more accurate and solid conclusions in the future.

In conclusion, we have reported for the first time that there were significant differences in the OS among previously untreated MBC patients with different GSTT1 and GSTM1 genotypes. Our results suggest that in addition to clinic-pathologic factors, genetic variants in GSTs might be suggestive factors in untreated MBC patients and further research is warranted.

## MATERIALS AND METHODS

### Patients

From March 2002 through November 2011, a total of 170 patients from Fudan University Shanghai Cancer Center (FUSCC) with previously untreated MBC were enrolled. Criteria for inclusion were as follows: female gender with histologically confirmed invasive ductal carcinoma, age of 18 to 70, with Eastern Cooperative Oncology Group (ECOG) performance status of 0 to 1, with adequate liver, renal function, and adequate bone marrow function. Exclusion criteria included: pretreatment of metastatic disease; more than one primary malignancy (except carcinoma *in situ* of the cervix or basal cell carcinoma of the skin with proper treatment); other serious complications/comorbidities that might affect survival.

Baseline information of these patients was collected and all specimens (blood samples) were obtained before treatment aiming at the metastatic disease. Classification of molecular subtypes and the clinic-pathology were based on the 2013 St. Gallen consensus [57]. Survival information was collected from hospital medical records and/or the follow-ups every 3 months. Each patient provided signed informed consent of using their DNA and clinical data. The study was approved by the Institutional Review Board of FUSCC.

### SNP genotyping

DNA was collected from 5-mL blood sample from each patient. The polymorphisms of multiple genes including GSTM1 null/present, GSTT1 null/present, and GSTP1 rs1695 were performed by PCR-based RFLP methods, then applied DNA sequencing of the PCR products to further confirm the genotypes [58]. To make sure the accuracy of method and total reproducibility, 15% random samples were genotyped repeat by different people.

### Statistical analysis

For each polymorphism, Pearson χ2 test was applied to test the Hardy-Weinberg equilibrium (HWE). OS was calculated from diagnosis of MBC to death. Survival distributions were analyzed by the Kaplan-Meier method and log-rank test was used to compare the survival analyses. Multivariate Cox proportional hazards models were applied to evaluate the effect of prognostic and clinical factors on OS, including age, molecular subtype, menstruation status, previous adjuvant treatment, relapse-free interval (RFI), number of metastatic sites, and type of metastatic site. Statistical significance was set at a level of 0.05 and all the statistical analyses were conducted using the SPSS software package (version 17. 0).

## Abbreviations

GSTM1: glutathione S-transferase theta
GSTP1: glutathione S-transferase pi
GSTT1: glutathione S-transferase mu
MBC: metastatic breast cancer
OS: overall survival
HR: hazard ratio
GST: glutathione S-transferase
T2DM: type 2 diabetes mellitus
RFI: relapse-free interval
ER: estrogen receptor
PR: progestrone receptor
HER2: human epidermal growth factor receptor 2
FUSCC: Fudan University Shanghai Cancer Center
ECOG: Eastern Cooperative Oncology Group
